# Factors Associated with Mortality Risk in Patients with Cardiogenic Shock Post-ST-Elevation Myocardial Infarction: Insights from a Regional Centre in Northwest Romania

**DOI:** 10.3390/medicina61040725

**Published:** 2025-04-14

**Authors:** Călin Florin Pop, Camelia Alexandra Coadă, Mihai Lupu, Ioan Florin Ferenț, Roxana Ioana Hodas, Andreea Pintilie, Mădălina-Ştefana Ursu

**Affiliations:** 1 Department of Cardiology, “Constantin Opriş” Emergency County Hospital, 430031 Baia Mare, Romania; medicbm@yahoo.com (C.F.P.);; 2 Faculty of Nursing and Health Sciences, University of Medicine and Pharmacy “Iuliu Hațieganu”, 400012 Cluj-Napoca, Romania; 3 Department of Morpho-Functional Sciences, University of Medicine and Pharmacy “Iuliu Hațieganu”, 400006 Cluj-Napoca, Romania

**Keywords:** myocardial infarction, mortality, cardiogenic shock, in-hospital mortality, ischaemic heart disease

## Abstract

*Background and Objectives*: ST elevation myocardial infarction (STEMI), particularly when complicated by cardiogenic shock (CS), is a critical condition associated with high mortality rates. Identifying predictors of in-hospital mortality can enhance patient management and outcomes. *Materials and Methods*: This observational, retrospective case–control study included STEMI patients, both complicated and uncomplicated by CS. Additionally, demographics, clinical characteristics, laboratory data and in-hospital mortality rates were analysed for STEMI patients with CS and those without CS. *Results*: This study included a total of 101 patients with STEMI, of whom 51 (50.5%) had STEMI without CS and 50 (49.5%) had STEMI with CS. No significant differences were observed in demographic characteristics or STEMI risk factors between the two groups. Emergency coronarography was performed in 90.1% of the patients, with successful thrombolysis achieved in 24.5%. Patients with CS exhibited a significantly higher mortality (52%) than those without CS (11.76%). Univariate analysis identified white blood cell counts, CK-MB, CK levels, elevated creatinine and uric acid levels and a reduced left ventricular ejection fraction (LVEF) as predictors of mortality. Logistic regression analysis revealed that LVEF and CK-MB were independent predictors of in-hospital mortality in patients with STEMI and CS. Each 1% increase in LVEF was associated with a reduced mortality risk (HR = 0.89; 95% CI 0.81–0.98; *p* = 0.018), while elevated CK-MB levels were linked to an increased mortality risk (HR = 1; 95% CI 1–1.01; *p* = 0.014). *Conclusions*: Reduced systolic function and elevated CK-MB levels are key predictors of in-hospital mortality and outcomes in STEMI patients with CS. These findings underscore the importance of early identification and support the development of targeted management strategies aimed at improving outcomes in this high-risk population.

## 1. Introduction

STEMI is a life-threatening medical emergency that exhibits variability across different countries and regions. In North America, approximately 965,000 cases of STEMI are reported annually. In Europe, the rates range from 80 to 370 cases per 100,000 person-years, while in Asia, it varies from 33 to 138 cases per 100,000 person-years [1]. The mortality rate associated with STEMI exceeds 15% with standard treatment; however, a decreasing trend has been observed since the implementation of thrombolysis, particularly with primary percutaneous coronary intervention (pPCI), which reduces 30-day mortality rates to between 2.7% and 8% [2]. CS is a critical condition characterised by low cardiac output and inadequate blood flow to peripheral tissues due to cardiac dysfunction, potentially leading to organ failure and death [3]. CS is associated with a high short-term mortality rate, primarily due to acute myocardial ischaemia caused by the occlusion of a coronary artery, resulting in the necrosis of cardiac myocytes [acute myocardial infarction (AMI)] and a marked deterioration of ventricular function [4,5]. CS complicates approximately 3–15% of AMI cases, with the highest incidence being 5–10% following STEMI. It is associated with 30-day and one-year mortality rates exceeding 40% and 50%, respectively, highlighting the critical need for improved risk stratification and management strategies [6,7,8].

The main treatment that has substantially improved outcomes for CS in patients with AMI is urgent coronary revascularisation. However, if pPCI is not performed promptly, mortality rates rise significantly. The advantages of reperfusion therapy are primarily linked to the rapid restoration of normal blood flow in the infarct-related artery, typically characterised as thrombolysis in myocardial infarction (TIMI) 3 flow. When the post-interventional TIMI flow is ≤2, it is strongly associated with worse outcomes, both during hospitalisation and up to six months after discharge [9,10,11].

Twenty-six years have passed since the SHOCK trial demonstrated that early revascularisation significantly reduced six-month mortality from 63% to 50% [12]. During this period, neutral trials such as IABP-SHOCK II and ECLS-SHOCK failed to show any benefits of systematic mechanical support [intra-aortic balloon pump (IABP) and extracorporeal membrane oxygenation (ECMO)] compared to standard treatment [6,13]. The Danish-German Cardiogenic Shock trial (DANGER-SHOCK), published in 2024, was the first to demonstrate the superiority of mechanical support using the IMPELLA CP axial device in treating patients with AMI-CS. The mortality rate was 46% in the IMPELLA group compared to 59% in the standard care group. However, the population studied was highly selective, with only 360 patients recruited over a span of 10 years [14]. Several trials are currently underway to clarify the role of optimising pharmacological treatment or mechanical support in AMI-CS patients, while current guidelines recommend against routine mechanical circulatory support (MCS) for these individuals [15,16,17].

Several prognostic factors for mortality and CS development have been identified in STEMI patients. These factors include LVEF, biomarkers of myocardial injury, laboratory variables, renal function parameters, elevation of the ST segment in the aVR lead, radiological findings and management strategies [18,19,20]. However, the relative importance of these factors, particularly in the context of CS, may vary across diverse studies, populations and healthcare systems. Therefore, the applicability and accuracy of different predictors in current practice remain uncertain, especially in resource-limited settings like Romania and similar countries. In a report from 2015, it was noted that, within a population of around 21.5 million people, there were 20 centres capable of undertaking pPCIs, 24 units that implanted cardiac rhythm devices and three centres that performed percutaneous interventional valve replacement techniques. There were also 13 cardiac surgery units. Mechanical support for CS and advanced heart failure (except IABP) was possible in only three centres [21]. This situation has not changed substantially in recent years, except for the emergence of private clinical cardiology departments and interventional procedure facilities concentrated in the four to five largest cities in the country [22]. In this context, a comprehensive understanding of the most relevant predictive factors is crucial for better identifying individual trajectories for high-risk STEMI patients.

In Romania, where the burden of cardiovascular disease is substantial, there is a paucity of recent, comprehensive studies examining prognostic factors in STEMI patients, particularly those complicated by CS. Before 2009, the main reperfusion strategy for STEMI patients was in-hospital thrombolysis, with approximately 40% of patients receiving thrombolytics. Less than 5% were treated with pPCI, and more than 50% of patients did not receive any reperfusion procedure. Consequently, in-hospital mortality remained high (around 13.5%) for the period 2000–2009. A national programme for pPCI in STEMI patients was initiated in Romania in 2010, and by 2012, the percentage of those treated with pPCI surpassed 50%. This change resulted in a substantial reduction in mortality, decreasing to around 7.28% in pPCI centres, while mortality remained at 14.20% in centres without pPCI facilities [23]. The first Romanian registry for STEMI (RO-STEMI) reported an incidence of CS at 6.6% with a mortality rate of 20.3% (probably underestimated, as it was not directly reported) [24]. The most recent multicentre study in Romania was conducted nearly a decade ago, focusing on heart failure of various aetiologies rather than specifically on STEMI with CS [25].

This study aimed to address the existing knowledge gap by evaluating the prognostic value of traditional risk factors, blood parameters and imaging data in predicting in-hospital mortality among STEMI patients, particularly those who develop CS, in a regional centre in northern Romania. By focusing on a specific geographic area and patient population, we sought to provide insights that could improve risk stratification and guide management strategies for this high-risk patient group. Additionally, we aimed to assess the impact of admission time relative to symptom onset on in-hospital cardiac mortality and morbidity.

## 2. Materials and Methods

### 2.1. Study Design and Patient Population

This was a single-centre, retrospective, observational case–control study evaluating patients from the Cardiology and Cardiac Intensive Care Unit wards at the Emergency County Hospital in Baia Mare, Maramureș, Romania. We reviewed cases of patients admitted with a diagnosis of STEMI from two counties in northern Romania during the period between January 2023 and October 2023. The County Hospital serves as the regional centre for immediate, invasive, catheter-based myocardial revascularisation for a population of up to 800,000 inhabitants across the two counties of Maramureș and Satu Mare [26].

### 2.2. Study Groups

After reviewing all patients with CS and STEMI (the case group), we matched an appropriate number of control cases consisting of STEMI patients without CS (the control group). For each case with CS, one control was selected from the STEMI patients who were hospitalised in our clinic at the same time. Cases and controls were individually matched based on gender, age (±2 years), ECG topography, and place of residence (rural/urban). All selected controls and cases were identified and reviewed around the same date and evaluated for risk factors as well as cardiovascular and metabolic comorbidities. We used the latest criteria from the Shock Academic Research Consortium (SHARC) expert panel to define CS: a systolic blood pressure (SBP) below 90 mm Hg for more than 30 min, the need for inotropes, vasopressors or MCS to maintain adequate blood pressure, and evidence of systemic hypoperfusion, such as cold or clammy extremities, altered mental status, elevated arterial lactate, and liver or kidney failure [27].

Two expert cardiologists from the team reviewed and double-checked the cases of patients with a working diagnosis of STEMI who were admitted <48 h prior. The diagnosis was considered valid if the patient presented with typical chest pain and ST elevation in any two contiguous leads of the electrocardiogram (ECG) performed during the first medical contact (FMC) for suspected acute coronary syndrome (ACS). The ICD-10-CM (codes I21.0-3) classification was used to extract data from the medical system employed for patient data management. For inclusion, the medical file had to contain data on the ECG, symptoms, haemodynamic status and any cardiac necrosis biomarkers (hs-cTn/CK/CK-MB) and/or invasive coronary angiography status. A variation in hs-cTn levels above the 99th percentile of a healthy population supports the diagnosis of AMI, according to the fourth universal definition of this condition [28]. The following ECG criteria were used for diagnosing STEMI: (1) new ST-segment elevation occurs at the J point in two contiguous leads, with a threshold >0.1 mV in all leads except V2 and V3; (2) in leads V2 and V3, the threshold is >0.2 mV for men older than 40, >0.25 mV for men under 40 and >0.15 mV for women [28]. Patients aged 18 or younger, as well as those with other forms of myocardial infarction (i.e., non-ST-elevation myocardial infarction/NSTEMI, second AMI, type 2 AMI, identified if any precipitating condition was mentioned in the clinical file, type 3, identified post-mortem and types 4–5 MI, linked to medical procedures), were excluded from the analysis.

### 2.3. Data Collection

Medical charts were assessed retrospectively to include baseline characteristics, risk factors and past medical history, which encompassed cardiac ischaemic disease, hypertension, obesity, dyslipidaemia, diabetes, chronic kidney disease and smoking status. Additionally, the assessment included ECG characteristics, timing of admission, interventional/pharmacological reperfusion status and biochemical data based on blood samples drawn within a time window of +/− 2 h of admission. LVEF was obtained through ultrasound evaluation during admission and hospitalisation. The results of reperfusion therapy, and the presence of mechanical and electrical complications of MI—including conduction disturbances such as Mobitz II or third-degree atrioventricular block and rhythm disturbances such as atrial fibrillation/atrial flutter, ventricular fibrillation and ventricular tachycardia—were also documented, along with in-hospital cardiovascular mortality.

All transthoracic echocardiograms (TTEs) were performed by expert cardiologists. In most cases, LVEF was first evaluated qualitatively in all of the patients received in the Emergency Unit, typically using a visual assessment (eyeball EF). Subsequently, LVEF was measured within the first 24–48 h in the Intensive Coronary Care Unit (ICU) using Simpson’s biplane method or the Teicholz quantification method in M-mode, or estimated visually when image quality did not permit accurate quantification. The American Society of Echocardiography recommends that the biplane-disc method be used to evaluate LVEF [29]. However, the American Society of Intensive Care Medicine indicates that LVEF can be qualitatively evaluated in emergency settings [30]. All TTE findings were recorded on a standardised echocardiography template. To reduce bias due to poor reproducibility, we selected and analysed the first LVEF recorded by Simpson’s biplane method or visually only if the absolute value of LVEF did not differ by more than 3% between the two methods [31].

Institutional Ethics Committee approval was obtained (registration no. 33657, date: 17 December 2024). The study was a retrospective, anonymised case–control study, with patients from the ICU department, where consent forms are not readily available or usable in a time-effective manner. Therefore, written consent was not obtained in the gross majority of cases. However, all the data were taken by the study investigators, in charts filled by the same authors. Furthermore, the study did not involve any additional procedures, modified treatments or management in all cases reviewed. All data were anonymised.

### 2.4. Study Endpoints

The study’s primary objective was to evaluate the prognostic ability of traditional risk factors, blood parameters and imaging data to predict the incidence of cardiac complications, CS and cardiac mortality. Secondary objectives included assessing in-hospital cardiac mortality and morbidity concerning the timing of admission relative to the onset of cardiac symptoms. Post-discharge mortality was not analysed.

### 2.5. Definitions and Variables

In-hospital cardiac mortality was defined as death due to cardiac causes occurring during the period of admission.

Mechanical complications were defined as acute mitral regurgitation resulting from papillary muscle rupture, a ventricular septal defect, pseudoaneurysm and ventricular free wall rupture.

The use and type of reperfusion therapy (pPCI or fibrinolysis) were noted throughout the entire hospital stay. Failure of fibrinolysis was defined as persistent or worsening chest pain, haemodynamic or electrical instability or electrocardiographic markers of failed reperfusion (<50% resolution of ST-segment elevation) occurring 60–90 min after the administration of pharmacological reperfusion.

Blood samples were collected from patients upon admission and as needed, during their hospitalisation. These samples were immediately transported to the laboratory for analysis. Clinical biochemistry testing was conducted using the Abbott ARCHITECT c8000 clinical chemistry analyser (Abbott, Illinois, United States), while clinical haematology testing was performed using the Mindray BC-6800 Plus analyser (Mindray, Shenzhen, China).

### 2.6. Statistical Analysis

All statistical analyses were performed using R version 4.4.0 (2024-04-24 UCRT; Puppy Cup) [32]. Continuous variables were reported as median with first and third quartiles. Normality was assessed using the Kolmogorov–Smirnov test. Statistical differences between groups were evaluated using either T-tests or Mann–Whitney tests, as appropriate. Categorical variables were reported as frequencies and percentages, with statistical differences between groups assessed using the Chi-Square test or Fisher’s exact test, as appropriate. Both univariable and multivariable logistic regression analyses were conducted to identify risk factors associated with in-hospital mortality. Relevant variables that were significant in the univariable analysis were included in the multivariable model to ascertain their independent association with the outcome. Receiver operating characteristic (ROC) curves were constructed, and the Youden index was used to obtain the optimal threshold for the variables associated with patient outcomes [33]. The effect size for the variables of interest was estimated using Cohen’s coefficient [34], while the study power was computed for a significance level of 0.05 [35]. A *p*-value of <0.05 was considered statistically significant.

## 3. Results

### 3.1. Characteristics of Patients with STEMI and CS

During the study period, we identified 529 patients with ACS, 104 with unstable angina (UA), 162 with NSTEMI and 263 patients with STEMI, of whom 50 developed CS (19.01%). After reviewing all patients with CS and STEMI (50 patients), we matched them to an appropriate number of control cases consisting of STEMI patients without CS. In total, 101 patients with STEMI were included, of which 51 (50.5%) had STEMI without CS (controls) and 50 (49.5%) had STEMI with CS (cases). The general characteristics of the groups are presented in Table 1. Briefly, there were no significant differences in demographic characteristics or major STEMI risk factors between the two study groups. A total of 91 patients (90.1%) underwent emergency coronarography, and 32 patients (31.7%) received thrombolysis, with a success rate of 78.1% (25/32 patients). The most frequently affected cardiac territory was the anterior wall, involved in 47 patients (46.5%), followed by the posterior wall in 28 patients (27.7%) and the inferoposterior wall in 8 patients (7.9%). The in-hospital mortality rate was 11.76% among STEMI patients without CS and 52% among those with CS. Patients who experienced in-hospital death developed arrhythmias more frequently (*p* < 0.001) and were more likely to develop CS (*p* < 0.001) (Supplementary Table S1).

All patients underwent routine bloodwork analyses, which included haematological and biochemical examinations (Table 1). Haemoglobin levels were slightly lower in patients with STEMI and CS, with borderline significance (*p* = 0.051). Conversely, WBCs were significantly higher (*p* = 0.001). CK-MB and, consequently, CK values were significantly higher in patients with STEMI and CS (*p* = 0.001 and *p* = 0.004, respectively), while hs-cTnI levels were comparable among all patients (*p* = 0.729). Kidney function, as revealed by creatinine and uric acid levels, was significantly altered in patients with STEMI and CS (*p* < 0.001 and *p* = 0.001, respectively). Systolic performance, evaluated by LVEF, was significantly lower in patients with STEMI and CS (*p* = 0.014). LVEF was estimated using Simpson’s method in 50% of the patients, while the remaining patients were evaluated visually.

Regarding the TIMI flow after percutaneous revascularisation, 20.5% of the patients exhibited a TIMI flow below three after pPCI, with no significant difference between the groups (*p* = 0.419). The pPCI was performed within 12 h of symptom onset for 73.27% of all patients, again showing no significant difference between the groups (*p* = 0.347). Among STEMI patients, those with CS received thrombolysis less frequently (26.53%) compared to those without CS (38%), although this difference did not reach statistical significance (*p* = 0.222).

### 3.2. Factors Associated with the Likelihood of In-Hospital Mortality of CS Patients

A total of 32 patients (31.7%) died during hospitalisation (Supplementary Table S1), of which 26 (81.25%) had CS (Table 2). Three patients with CS were treated unsuccessfully with IABP, which was the only mechanical support available.

A comparison of the CS survivors with those who died in the hospital revealed significant differences in the development of CS. In terms of biochemical data, WBC (*p* = 0.004), CK levels (*p* < 0.001), CK-MB (*p* < 0.001), glycaemia (*p* < 0.001), and creatinine (*p* < 0.001) and uric acid levels (*p* = 0.012) were all significantly higher in patients who experienced in-hospital death. Conversely, LVEF was significantly lower in these patients (*p* < 0.001). A pPCI procedure was performed in 88% of patients, with no significant difference between those who died and the survivors (*p* = 0.376). Regarding the TIMI flow after percutaneous revascularisation, 33.33% of the CS patients who died had a TIMI flow below three (*p* = 0.388). However, there were no significant differences when compared to those who survived (Table 2). The pPCI was performed within 12 h of symptom onset for 64% of the patients with CS, with no difference between those who died and the survivors (*p* = 0.388). Only 13 out of 50 patients with STEMI and CS (26%) received thrombolysis before pPCI, with no significant difference between survivors and those who died.

We included relevant variables in the logistic regression model, namely LVEF, glycaemia and CK-MB. In both univariable and multivariable models, LVEF and CK-MB remained independent predictors of in-hospital mortality in patients with STEMI and CS. Specifically, patients with higher LVEF values exhibited a lower risk of in-hospital death, with each percentage increase in systolic function correlating with a decreased risk of this adverse outcome (HR = 0.89; 95% CI 0.81–0.98; *p* = 0.018). Conversely, higher CK-MB levels were associated with an increased risk of in-hospital mortality, suggesting that each unit increase in CK-MB slightly elevated the risk of death (HR = 1; 95% CI 1–1.01; *p* = 0.014) (Table 3).

### 3.3. Defining the Cut-Off for Increased Risk of Mortality of Patients with STEMI and CS

**Figure 1:**
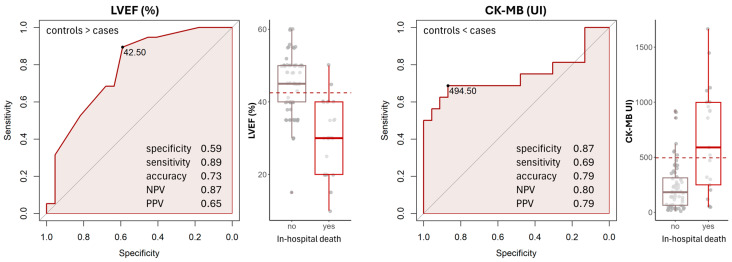
ROC curves for the variables found to significantly impact patient’s in-hospital death. The point on the ROC curve indicates the best cut-off threshold for discrimination defined by the Youden index. Dotted lines on the boxplots present the cut-off thresholds. LVEF: left ventricle ejection fraction; NPV: negative predictive value; PPV: positive predictive value.

Youden’s J index was calculated for each coordinate of the ROC curve. The coordinate with the highest J index was used to determine the optimal cut-off values for LVEF and CK-MB. The cut-off for increased mortality risk was identified as an LVEF of 42.5%, although this threshold exhibited intermediate specificity at only 60%. The CK-MB threshold for increased risk was determined to be 494.5 U/L, with a specificity approaching 90% (Figure 1). Both LVEF and CK-MB demonstrated similar performance indices (DeLong test *p*-value = 0.917), with modest accuracies of 0.73 and 0.79, respectively, in predicting patient mortality (Figure 1). When analysing all patients included in the study, the same variables continued to serve as predictors of in-hospital death (Supplementary Table S2).

## 4. Discussion

This study, which included STEMI patients with CS (49.5%) and without CS (50.5%), aimed to evaluate routine paraclinical investigations and the risk of in-hospital mortality in these patients. To optimally analyse whether classical or additional variables or prognostic factors were present among STEMI patients who developed CS, we selected control patients from among first-STEMI patients. This is a single-centre retrospective study with a low level of evidence; however, conducting a prospective study is challenging due to the particularities and ethical considerations surrounding CS. Nevertheless, this study remains valuable, as there are existing examples in the literature [36,37].

### 4.1. Results in the Context of Published Literature

The previous literature and clinical studies have elaborated on various risk scores, including prognostic factors for in-hospital mortality in STEMI patients. The IABP-SHOCK II risk score [6] and the CardShock score [18] identify risk factors in the form of regression scores that can be used for mortality prediction. LVEF is a well-known predictor in STEMI patients and has also been included in mortality scoring systems such as the ACEF (age, creatinine and ejection fraction) [38]. In our study, LVEF and CK-MB were identified as independent predictors of in-hospital mortality. The cut-off for LVEF, ≤42.5% (Figure 1), which predicts elevated mortality and CS development, was higher than expected based on other studies (≈33–35%) but exhibited intermediate specificity at only 60% [18,19,39]. However, this value is consistent with findings from a study indicating that patients with an LVEF <40% had a 30-day mortality rate six times higher than those with an LVEF >40% [39]. The CK-MB threshold for an increased risk of developing CS was determined to be 494.5 U/L, with a specificity approaching 90%, strongly suggesting significant myocardial damage [40]. Therefore, these findings highlight the prognostic importance of baseline values of systolic function and myocardial injury in the outcomes of patients with STEMI and/or CS.

Although there are multiple methods for assessing LVEF via TTE, our study initially involved a visual estimation of LVEF in the patients. In this context, the eyeball LVEF differed by <3% compared to Simpson’s method of discs, which was typically performed 24–48 h later [31,41,42]. Therefore, as outlined in our protocol, these values were analysed. Our study’s eyeball LVEF data align with findings from a study conducted by Gudmundsson et al., which demonstrated that qualitative estimation of ejection fraction closely correlated with all formal methods, and the correlation coefficient improved with the reliability of the formal method [43].

CS is recognised as the most severe complication in patients with STEMI. The mortality rate among patients with STEMI and CS has remained relatively high despite various improvements in the management and treatment of these patients [6,7,8,44]. In our study, the in-hospital mortality rate for patients with STEMI and CS was 52%, compared to 11.76% for those with STEMI without CS. This finding aligns with other studies that report similar mortality rates [12,13,45]. Although some studies indicate that index hospitalisation is the most critical period for STEMI-CS patients, the absence of a long-term follow-up in our study may have led to us overlooking other important factors related to patient mortality [6,45]. Our data are consistent with existing evidence and suggest that the initial treatment of these patients is essential not only for immediate survival but also for mortality rates at one month and one year [46,47]. Nevertheless, after 30 days, the mortality curves remained elevated but began to flatten thereafter [6,12,13,48].

Research has demonstrated that acute kidney injury, which develops in up to 50% of patients with CS, is a significant predictor of poor outcomes, irrespective of the underlying cause of CS [18,19,46,49,50]. Various studies have shown a significant association between creatinine and uric acid levels and CS prognostics at the time of admission or during the ICU stay [51,52,53]. In our data, creatinine and uric acid levels were significantly higher in STEMI patients with CS compared to those without CS, and were also associated with in-hospital mortality. However, in the multivariable analysis, this significance diminished, with only CK-MB and LVEF remaining as independent predictors of patient outcomes. It is important to note that our analysis used baseline measurements, which may explain the lack of statistical association. Acute kidney injury develops progressively over time due to tissue hypoperfusion, which might not be fully reflected in the initial patient admission data. Additionally, the single-centre and retrospective observational study design may have influenced the results due to both measured and unmeasured confounding variables; for example, there was no evidence of pre-admission creatinine levels, and biomarkers like NGAL and CysC were not measured during the hospital stay [54].

Some studies have suggested that ST elevation in aVR (aVR ↑ST) is an independent predictor of CS and left main coronary artery disease [20,55]. A greater magnitude of ST elevation has been associated with augmented mortality and a higher incidence of heart failure, reinfarction, or CS [56]. In our study, we analysed aVR ↑ST > 0.1 mV and found that the frequencies (around 20%) were not significantly different between STEMI patients with and without CS and between CS patients who survived and those who did not, as shown in Table 1 and Table 2. The rate of aVR ↑ST >0.1 mV was 28% in a study involving 210 consecutive, unselected patients from September 2002 to 2006 with CS due to AMI [20]. Therefore, based on our findings, we cannot establish the prognostic significance of aVR ↑ST > 0.1 mV, as our study had a limited number of cases, and we were highly selective of the control patients.

Most patients (74 out of 101, 73%) were admitted to our centre <12 h after the onset of symptoms, and 55 of these patients (54.5%) were transferred from a non-PCI facility, as the detrimental effects of delays in treatment initiation for these patients are well established [11,57]. However, our record-keeping system did not permit the identification of door-to-balloon and door-to-needle times. While only 31% of patients (32 out of 101) received thrombolysis in the hospital, there was a notable difference, in favour of those without CS (38% vs. 26%; *p* = 0.376). This trend of administering pre-transfer thrombolysis less frequently than expected was also noted at the national level after the initiation of the pPCI programme [23]. We observed that most patients, with very few exceptions from the hospital city residence, had a median transfer time of around 380 min from non-PCI centres. Longer transfer times from first medical contact (FMC) and less frequent thrombolysis may help explain the high incidence of CS (19.01%) and the relatively elevated mortality rate (11.76%) among our STEMI patients. In our study, not only was the total ischaemic time > 6.3 h, but the objective of achieving revascularisation within 120 min from FMC was accomplished in <10% of patients who arrived directly at our hospital from their city of residence. The data are consistent with those from a recent prospective Romanian study on STEMI patients, which demonstrated the difficulties of prehospital care [58]. In that study, total ischaemic times frequently averaged around 6.4 h, with prehospital thrombolysis administered in only 6.4% of cases, and only 35.5% of patients were revascularized within the optimal time frame in Bucharest, the capital of the country [58]. Studies have shown that each additional minute from FMC to revascularisation increases the chances of lowering LVEF by 2% (95% CI: 1.004–1.041) and raises the risk of death by 2% (95% CI: 1.002–1.04) in STEMI patients [59]. Also, in our research, 37.04% of the patients who died had a TIMI flow of ≤2, and only 28.12% achieved complete revascularisation (Supplementary Data). This may also explain the high incidence of CS among our patients and correlates with the observation that the post-interventional TIMI flow of ≤2 was strongly associated with adverse outcomes during hospitalisation and six months following discharge [9,11]. When comparing the RO-STEMI registry data from 1997 to 2009, it was found that approximately 6.6% of STEMI patients developed CS. In the northwest region, which includes the counties surveyed in the present study, the incidence of CS was notably higher at 11.7%. Thrombolytics were used as the primary treatment approach in 37.07% of cases nationwide. Additionally, among patients experiencing CS and/or pulmonary oedema, the rate of thrombolytic use was 9.25% in the registry, while it was 26.53% in our study. In terms of pPCI, 58.34% of patients in the northwest region received this standard of care in 2011, while 90% of patients in our study underwent percutaneous intervention. This indicates an increase in guideline-directed therapy and the enhanced capacity of regional hospitals to offer specialised care [23,24]. Moreover, to further improve the care of these patients, the Romanian Ministry of Health has developed a national strategy for preventing cardiovascular and cerebrovascular diseases for the years 2024–2030 [60].

### 4.2. Study Limitations

Firstly, the data were observational, originating from a single regional centre and based on a registry, making it vulnerable to unmeasured confounding factors and selection bias. The angiographic results were reported by the site rather than being adjudicated by a core laboratory. However, the angiographic results are usually double-checked by our interventional cardiologists, which may help reduce interpretation bias.

The LVEF was first estimated visually for 50% of the patients, which may lead to imprecise data regarding echocardiographic parameters.

Secondly, our data are influenced by survivorship bias, as they only include patients with demographic characteristics and healthcare that enhance their likelihood of surviving until medical intervention.

Thirdly, during the study period, MCS devices, except for the IABP, were not used in our programme. Three patients with CS were treated with IABP, but the results were not statistically significant. Current guidelines recommend against the routine use of MCS in AMI-CS patients [17]. Our study primarily aimed to understand the outcomes of STEMI complicated by CS, treated mainly with pharmacological interventions within the specific Romanian healthcare context, characterised by limited resources. The systematic and efficient use of mechanical support in CS patients may be addressed by ongoing multicentre trials (e.g., the ISRCTN82431978 trial) [16].

In addition to the known limitations of case–control studies, an important weakness of this research was the small sample size, as well as the lack of long-term follow-up. Therefore, the findings of this study should be interpreted with caution for these reasons. However, existing research indicates that index hospitalisation represents the most critical period for STEMI-CS patients, during which the mortality rate remains high but subsequently flattens. This suggests that the initial treatment of these patients is essential, while the absence of long-term follow-up may have overlooked other important factors related to patient mortality.

Finally, as previously mentioned, our work is a single-centre retrospective study with a low level of evidence. However, conducting a prospective study is challenging due to the particularities and ethical considerations associated with CS.

### 4.3. Future Directions

Patients with STEMI, especially those complicated by CS, represent a heterogeneous population, and the results of different studies may not be routinely applicable in clinical practice. Internationally, ongoing randomised clinical trials are attempting to identify potential adjuvant pharmacological treatments and potential biomarkers for CS prediction and recovery and to establish appropriate criteria for the use of mechanical support. At the national level, there is a pressing need for consistent health policies to improve geographical access to interventional and cardiovascular surgical procedures. This includes upgrading existing cardiovascular units and hospitals with qualified staff and modern technologies to offer advanced cardiac care. Additionally, it is essential to improve the quality of care in current clinical practice by admitting high-risk AMI/STEMI patients with an LVEF of <42.5% and higher CK-MB levels to tertiary centres capable of providing more aggressive pharmacological and mechanical support.

## 5. Conclusions

Patients with CS exhibited higher in-hospital mortality rates. Lower LVEF, elevated glycaemia, CK-MB, creatinine levels and the development of rhythm disorders were associated with adverse patient outcomes. An LVEF (below 42.5%) and a CK-MB (above 494.5 U/L) were identified as independent mortality predictors. These parameters can aid physicians in the early identification of high-risk patients during their STEMI evolution, enabling the implementation of more intensive pharmacological or mechanical support strategies, particularly in resource-limited settings.

## Figures and Tables

**Table 1:** General, demographic and laboratory characteristics of the patients.

Variable		Patients with STEMI N = 51	Patients with STEMI and CS N = 50	*p*-Value
Age at presentation, median (IQR)		65 (51.5; 73)	68.5 (58; 75.75)	0.115
Sex, N (%)	Female	18 (35.29)	19 (38)	0.778
	Male	33 (64.71)	31 (62)	
Smoker status, N (%)		17 (33.33)	18 (42.86)	0.345
Arterial hypertension, N (%)		27 (52.94)	31 (64.58)	0.240
Diabetes mellitus, N (%)		16 (31.37)	10 (20.83)	0.234
Number of affected coronary vessels, N (%)	1	16 (34.04)	14 (31.82)	0.505
	2	11 (23.4)	15 (34.09)	
	3	20 (42.55)	15 (34.09)	
History of ischemic cardiomyopathy, N (%)		3 (6.38)	9 (20.45)	**0.047**
Time from symptom debut (h), N (%)	<12	42 (84)	32 (76.19)	0.347
	>12	8 (16)	10 (23.81)	
Transferred from another hospital, N (%)		29 (56.86)	26 (53.06)	0.702
Thrombolysis, N (%)		19 (38)	13 (26.53)	0.222
TIMI, N (%)	≤2	9 (20)	12 (27.27)	0.419
	3	36 (80)	32 (72.73)	
aVR ↑ST ≥1 mm, N (%)		10 (19.6)	10 (20)	0.960
**MI complications**				
Mechanical complications, N (%)		1 (1.96)	0 (0)	1
Arrhythmia, N (%)		3 (5.88)	24 (48.98)	**<0.001**
Heart block, N (%)		5 (9.8)	10 (20)	0.150
In-hospital death, N (%)		6 (11.76)	26 (52)	**<0.001**
**Paraclinical investigations**				
Haemoglobin (g/dL), median (IQR)		14 (12.8; 15)	13.1 (11.6; 14.3)	0.051
WBC (/μL), median (IQR)		10,770 (9400; 13,350)	14,700 (10,700; 18,675)	**0.001**
CRP (mg/L), median (IQR)		17 (7.3; 75)	45.17 (13.77; 143.5)	0.123
hs-cTnI (ng/L), median (IQR)		7000 (431; 37,433.5)	18,434 (189.5; 32,437.5)	0.729
CK (U/L), median (IQR)		943.5 (398; 2443)	2033 (1211; 4275)	**0.004**
CK-MB (U/L), median (IQR)		142 (58; 295)	320 (192; 739.5)	**0.001**
Glycemia (mg/dL), median (IQR)		109 (96; 159)	126 (107; 194)	0.406
LDL (mg/dL), median (IQR)		97.6 (80.5; 139.6)	80.2 (61; 110)	**0.025**
Creatinine (mg/dL), median (IQR)		0.91 (0.76; 1.13)	1.47 (1; 2.07)	**<0.001**
Uric acid (mg/dL), median (IQR)		6.95 (5.5; 7.88)	8.2 (6.4; 10.3)	**0.001**
LVEF (%), median (IQR)		45 (40; 50)	38 (30; 48)	**0.014**

IQR: interquartile range; N: number of cases; STEMI: ST elevation myocardial infarction; ↑ST—ST segment elevation; CS: cardiogenic shock; aVR ↑ST—ST segment elevation in lead aVR; WBC: white blood count; CRP: C reactive protein; hs-cTnI: high sensitivity troponin; CK: creatine kinase; CK-MB: creatine kinase myocardial bound; LDL: low-density lipoprotein. Bolded *p*-values are significant.

**Table 2:** Characteristics of patients with STEMI and cardiogenic shock grouped based on their survival status.

Variable		Survivors N = 24	In-Hospital Death N = 26	*p*-Value
Age, median (IQR)		72 (61.75; 75.75)	63 (56.25; 74.75)	0.127
Sex, N (%)	Female	12 (50)	7 (26.92)	0.093
	Male	12 (50)	19 (73.08)	
Smoker status, N (%)		8 (38.1)	10 (47.62)	0.533
Arterial hypertension, N (%)		16 (69.57)	15 (60)	0.489
Diabetes mellitus, N (%)		5 (20.83)	5 (20.83)	1
Obesity, N (%)		4 (18.18)	3 (13.04)	0.699
Dyslipidemia, N (%)		12 (66.67)	9 (50)	0.310
CKD, N (%)		5 (25)	2 (10.53)	0.407
Number of affected coronary vessels, N (%)	1	6 (26.09)	8 (38.1)	0.381
	2	7 (30.43)	8 (38.1)	
	3	10 (43.48)	5 (23.81)	
History of ischemic cardiomyopathy, N (%)		5 (21.74)	4 (19.05)	1
Time from symptom debut (h), N (%)	<12	18 (81.82)	14 (70)	0.477
	>12	4 (18.18)	6 (30)	
Transferred from another hospital, N (%)	no	9 (37.5)	14 (56)	0.195
	yes	15 (62.5)	11 (44)	
Thrombolysis, N (%)	no	19 (79.17)	17 (68)	0.376
	yes	5 (20.83)	8 (32)	
TIMI, N (%)	≤2	5 (21.74)	7 (33.33)	0.388
	3	18 (78.26)	14 (66.67)	
aVR ↑ST ≥1 mm, N (%)		5 (20.8)	5 (19.2)	0.880
**MI complications**				
Arrhythmia, N (%)		9 (37.5)	15 (60)	0.115
Heart block, N (%)		7 (29.17)	3 (11.54)	0.119
**Paraclinical investigations**				
Haemoglobin (g/dL), median (IQR)		12.8 (11.45;13.93)	14 (11.8; 14.3)	0.096
WBC (/μL), median (IQR)		13,650 (10,450; 15,820)	17,000 (11,675; 23,420)	0.240
CRP (mg/L), median (IQR)		33.91 (13.24; 121.88)	47.67 (19.35; 207.25)	0.286
hs-cTnI (ng/L), median (IQR)		18,434 (254.5; 30,125)	12,689.5 (429.5; 32,437.5)	0.887
CK (U/L), median (IQR)		1814 (691.5; 2056.25)	5165 (2252; 6360)	**<0.001**
CK-MB (U/L), median (IQR)		274 (157.5; 386)	887 (237.25; 1024.75)	**0.009**
Glycemia (mg/dL), median (IQR)		111 (98.5; 167.5)	159.5 (122.25; 195)	**0.035**
LDL (mg/dL), median (IQR)		79.1 (54.35; 105.5)	87 (70; 122)	0.462
Creatinine (mg/DL), median (IQR)		1.22 (0.9; 2.08)	1.9 (1.27; 2.06)	0.097
Uric acid (mg/dL), median (IQR)		7.95 (6.4; 10.3)	8.2 (7.2; 9.8)	0.723
LVEF (%), median (IQR)		45 (35; 50)	30 (22.5; 40)	**0.002**

IQR: interquartile range; N: number of cases; STEMI: ST elevation myocardial infarction; aVR ↑ST—ST segment elevation in lead aVR; CKD: chronic kidney disease; WBC: white blood count; CRP: C reactive protein; hs-cTnI: high sensitivity troponin; CK: creatine kinase; CK-MB: creatine kinase, myocardial bound; LDL: low-density lipoprotein; LVEF: left ventricle ejection fraction. Bolded *p*-values are significant.

**Table 3:** Univariable and multivariable analysis of the variables associated with patient in-hospital death of STEMI patients with cardiogenic shock.

	Univariable Analysis		Multivariable Analysis	
Variable	HR (95% CI)	*p*-Value	HR (95% CI)	*p*-Value
LVEF (%)	0.90 (0.84; 0.97)	**0.005**	0.89 (0.81; 0.98)	**0.018**
CK-MB	1 (1; 1.01)	**0.005**	1 (1; 1.01)	**0.014**
Glycemia	1.01 (0.99; 1.02)	0.148		

Bolded *p*-values are significant.

## Data Availability

All the data were taken by the study investigators, in charts filled by the same authors. Furthermore, the study did not involve any additional procedures, modified treat-ments or management in all cases reviewed. All data were anonymised.

## Supplementary Materials

The following supporting information can be downloaded at: https://www.mdpi.com/article/10.3390/medicina61040725/s1, Table S1: Characteristics of patients who died during hospitalization. Table S2: Univariable and multivariable analysis of the parameters associated with patient in-hospital death of STEMI patients.
