# Supplementary material for: Factors Associated with Mortality Risk in Patients with Cardiogenic Shock Post-ST-Elevation Myocardial Infarction: Insights from a Regional Centre in Northwest Romania
Source: Medicina (Kaunas). 2025 Apr 14;61(4):725. doi: 10.3390/medicina61040725 (PMC12029066; doi:10.3390/medicina61040725)
Supplement: Supplementary file 1 [file medicina-61-00725-s001.zip › medicina-3529172-supplementary.pdf]

Study power considerations

A posteriori power analysis was conducted for the primary aim of the study. For the variables of interest Cohen's d effect size was estimated and then used to assess the power of the study. When considering all patients included in the study, a power of 99% was obtained for the association of FEVS and CK-MB with in-hospital patient death. When considering only patients with STEMI and CS, a power of 96% was obtained for the same variables and their association with in-hospital patient death. For all power calculations a significance threshold of 0.05 was used.

Supplementary Tables

Supplementary Table 1. Characteristics of patients who died during hospitalization.

**Variable**		**Survivors** N=69	**Early (in-hospital) death** N=32	**p-value**
Age, median(IQR)		66(54;74)	67(57.75;76.25)	0.411
Sex, N(%)	Female	26(37.68)	11(34.38)	0.748
	Male	43(62.32)	21(65.62)	
Smoker status, N(%)	no	41(62.12)	17(62.96)	0.939
	yes	25(37.88)	10(37.04)	
Arterial hypertension, N(%)	no	29(42.65)	12(38.71)	0.712
	yes	39(57.35)	19(61.29)	
Diabetes mellitus, N(%)	no	51(73.91)	22(73.33)	0.952
	yes	18(26.09)	8(26.67)	
Obesity, N(%)	no	52(77.61)	26(89.66)	0.165
	yes	15(22.39)	3(10.34)	
Dyslipidemia, N(%)	no	24(38.71)	10(45.45)	0.580
	yes	38(61.29)	12(54.55)	
CKD, N(%)	no	55(84.62)	21(84)	1
	yes	10(15.38)	4(16)	
Number of affected coronary vessels, N(%)	1	21(32.81)	9(33.33)	0.418
	2	16(25)	10(37.04)	
	3	27(42.19)	8(29.63)	
History of ischemic cardiomyopathy, N(%)	no	57(89.06)	22(81.48)	0.330
	yes	7(10.94)	5(18.52)	
Time from symptom debut (hours), N(%)	<12	56(84.85)	18(69.23)	0.227
	12-24	5(7.58)	4(15.38)	
	>24	5(7.58)	4(15.38)	
Transferred from another hospital, N(%)	no	29(42.03)	16(51.61)	0.373
	yes	40(57.97)	15(48.39)	
Thrombolysis, N(%)	no	46(66.67)	21(70)	0.745
	yes	23(33.33)	9(30)	

TIMI, N(%)	<=2	11(17.74)	10(37.04)	0.050
	3	51(82.26)	17(62.96)	
**MI complications**				
Mechanical complications, N(%)	no	68(98.55)	32(100)	1
	yes	1(1.45)	0(0)	
Arrhythmia, N(%)	no	58(84.06)	15(48.39)	**<0.001**
	yes	11(15.94)	16(51.61)	
Heart block, N(%)	no	59(85.51)	27(84.38)	1
	yes	10(14.49)	5(15.62)	
Cardiogenic shock, N(%)	no	45(65.22)	6(18.75)	**<0.001**
	yes	24(34.78)	26(81.25)	
**Paraclinical investigations**				
Hemoglobin (g/dl), median(IQR)		13.5(12;14.8)	14(11.8;14.3)	0.774
WBC (/µl), median(IQR)		11700(9420;14200)	17000(11675;21725)	**0.004**
CRP (mg/l), median(IQR)		22(10.15;98.25)	47.67(18.65;115)	0.167
hs-cTnI (ng/l), median(IQR)		8877(171.25;34135.75)	7000(683;35207)	0.885
CK (U/l), median(IQR)		1008(448;2022.5)	4787(2462.5;6360)	**<0.001**
CKMB (U/l), median(IQR)		182(63.5;312.5)	589(249;998)	**<0.001**
Glycemia (mg/dl), median(IQR)		108.5(95;157.5)	177(122.25;228.75)	**<0.001**
LDL (mg/dl), median(IQR)		93.6(76;127.8)	95(70.75;144.45)	0.813
Creatinine (mg/dl), median(IQR)		0.98(0.8;1.25)	1.9(1.22;2.16)	**<0.001**
Uric acid (mg/dl), median(IQR)		6.95(5.75;8.1)	8.2(7.2;9.8)	**0.012**
LVEF (%), median(IQR)		45(40;50)	30(20;40)	**<0.001**

IQR: interquartile range; N: number of cases; STEMI: ST elevation myocardial infarction; CS: cardiogenic shock; CKD: chronic kidney disease; WBC: white blood count; CRP: C reactive protein; hsTn: high sensitivity troponin; CK: creatine kinase; LDL: low-density lipoprotein.

Supplementary Table 2. Univariable and multivariable analysis of the parameters associated with patient in-hospital death of STEMI patients.

	**Univariable analysis**		**Multivariable analysis**	
**Variable**	**HR(95%CI)**	**p-value**	**HR(95%CI)**	**p-value**
Cardiogenic shock	8.12(2.94;22.46)	**<0.001**	6.03(0.26;142.22)	0.265
LVEF (%)	0.85(0.79;0.92)	**<0.001**	0.89(0.79;0.99)	**0.042**
Arrhythmia	5.62(2.16;14.61)	**<0.001**	1.33(0.08;22.37)	0.842
Glycemia	1.01(1;1.02)	**0.002**	1.01(0.99;1.03)	0.254
CK-MB	1(1;1.01)	**<0.001**	1.01(1;1.01)	**0.003**
Creatinine	1.91(1.12;3.26)	**0.018**	1.4(0.8;2.44)	0.242
Uric acid	1.13(0.96;1.34)	0.143		
